# Portable Detection of Copper Ion Using Personal Glucose Meter

**DOI:** 10.3390/s24217002

**Published:** 2024-10-31

**Authors:** Bin Du, Taoying Chen, Anqi Huang, Haijun Chen, Wei Liu

**Affiliations:** 1 School of Food Science and Engineering, Guiyang University, Guiyang 550005, China; chentaoying_gyu@126.com (T.C.); huanganqi0804@163.com (A.H.); chenhaijun0415@163.com (H.C.); 2 Cancer Center and Department of Pharmacology and Toxicology, Medical College of Wisconsin, Milwaukee, WI 53226, USA

**Keywords:** personal glucose meter, copper ion, Fe_3_O_4_ magnetic nanoparticles, alkyne–azide cycloaddition reaction

## Abstract

A simple and sensitive method for Cu^2+^ detection was developed using the Cu^+^-catalyzed alkyne–azide cycloaddition reaction, Fe_3_O_4_ magnetic nanoparticles (MNPs) as the reaction platform, and a portable blood glucose meter (PGM) as the detection method. Gold nanoparticles (AuNPs) were labeled with glucose oxidase (GOx) and alkyne-functionalized, terminally thiolated ssDNA (C2). In the presence of Cu^2+^ and ascorbate, the functionalized AuNPs were captured onto MNPs modified with azide-functionalized ssDNA (C1) via the Cu^+^-catalyzed alkyne–azide cycloaddition reaction. The GOx on the AuNPs’ surface oxidized glucose (Glu) into gluconic acid and H_2_O_2_, reducing the Glu content in the reaction solution, which was quantitatively detected by the PGM. Under optimal conditions, the PGM response of the system showed a good linear relationship with the logarithm of Cu^2+^ concentration in the range of 0.05 to 10.00 μmol/L, with a detection limit of 0.03 μmol/L (3σ). In actual tap water samples, the spiked recovery rate of Cu^2+^ was between 92.30% and 113.33%, and the relative standard deviation was between 0.14% and 0.34%, meeting the detection requirements for Cu^2+^ in real water samples.

## 1. Introduction

Copper, apart from Fe^2+^ and Zn^2+^, ranks as the third essential trace element needed by humans. It plays a key role in reductases, participating in numerous oxidation–reduction reactions, and is vital for various physiological processes [1]. Adequate Cu^2+^ levels can promote the synthesis of hemoglobin, facilitate hematopoietic function, maintain the normal physiological functions of bones, blood vessels, and skin, boost metabolism, and enhance cellular immunity, among other benefits. Nevertheless, prolonged exposure to high levels of Cu^2+^-based toxins can result in gastrointestinal discomfort, as well as potential harm to the liver and kidneys, and may lead to various neurological disorders [2,3,4,5]. The World Health Organization (WHO), the United States Environmental Protection Agency (EPA), and the Chinese Ministry of Health have set the maximum allowable levels of Cu^2+^ in drinking water at 2 mg/L, 1.27 mg/L, and 1 mg/L, respectively [6]. Currently, methods for detecting Cu^2+^ primarily include stripping voltammetry [7], atomic absorption spectroscopy [8], photoelectrochemical methods [9], and fluorescence analysis [10]. While these methods exhibit good selectivity and high sensitivity, they are associated with expensive instruments, complex sample preparation, and high detection costs. Therefore, there is an urgent need to develop a rapid, sensitive, operationally simple, and high-throughput screening method for the real-time detection of Cu^2+^.

The personal glucose meter (PGM) serves as an optimal portable detection platform owing to its advantages of portability, short analysis time, low cost, ease of operation, and reliable quantitative results [11,12], and it has been applied in the detection of various analytes, including ions [13,14], small molecules [15], nucleic acids [16], proteins [17], etc. The Cu^+^-catalyzed alkyne–azide cycloaddition reaction entails the reaction between an alkyne and an azide group, resulting in the formation of a 1,2,3-triazole under the catalysis of Cu^+^ [18]. A trace amount of Cu^+^, produced through the reduction of Cu^2+^ in the presence of sodium ascorbate, serves as a catalyst, facilitating the efficient conjugation between the azide and alkyne groups, thereby providing a crucial copper-mediated signal for the detection of Cu^2+^ [19].

In this study, we have developed a method for the detection of Cu^2+^ using a portable glucose meter as the detection device. The approach utilizes MNPs as an efficient carrier for immobilizing ssDNA probes modified with alkyne, and gold nanoparticles (AuNPs) labeled with glucose oxidase (GOx) and alkyne-functionalized, terminally thiolated ssDNA were served as signal transduction labels. The recognition element for target Cu^2+^ is based on the Cu^+^-catalyzed azide–alkyne cycloaddition which results in the chemical ligation of ssDNA modified with alkyne and azide groups. Compared to other Cu^2+^ sensors, this method utilizes magnetic nanoparticles as efficient carriers for the bio-recognition element, allowing for the rapid isolation and enrichment of target molecules from complex samples, thereby reducing the interference effects on the detection signal. The utilization of a portable glucose meter as the detection tool offers advantages such as simplicity of operation, low cost, portability, and ease of use without the need for specialized training. This approach addresses the limitations of traditional instrumental analytical methods and presents a novel avenue for the development of portable Cu^2+^ detection devices.

## 2. Materials and Methods

### 2.1. Reagents and Apparatus

Sodium ascorbate, tris-(hydroxypropyltriazolylmethyl) amine (THPTA), HAuCl4·H2O, Triton X-100, 3-aminopropyltriethoxysilane (APTES), tetraethoxysilane (TEOS), Triton X100, Ammonium hydroxide, 1-ethyl-3-(3-dimethylaminopropyl) carbodiimide (EDC), and N-hydroxysulfosuccinimide sodium salt (sulfo-NHS) were purchased from Sigma-Aldrich (St. Louis, MO, USA). Sodium citrate, CuCl_2_·2H_2_O, FeCl_3_·6H_2_O, FeSO_4_·7H_2_O, and other metal salts were obtained from Sinopharm Chemical Reagent Co., Ltd. (Shanghai, China). GOx was purchased from Sangon Biotech (Shanghai, China). The oligonucleotides probe was synthesized by Sangon Biotech (Shanghai, China), and the sequences of the oligonucleotides were listed as follows: C1: 5′-COOH-TAGTCTGATTGC-azide-3′; C2: 5′-alkyne-ATCCTTATCAAT-SH-3′. A glucose meter (AG-3 Sinocare, Changsha, China) was used to determine the glucose concentration.

Transmission electron microscopy observations were carried out using the FEI Tecnai F20 microscope (Hillsboro, OR, USA). Particle size and Zeta potential were collected on a Zetasizer Nano ZS-90 (Malvern Instruments Ltd., Malvern, UK). The ultraviolet−visible absorption spectrum was measured using a UV-2600 ultraviolet spectrophotometer (Shimadzu, Kyoto, Japan). The magnetic property was analyzed by a LakeShore 7404 vibrating sample magnetometer (LakeShore, CO, USA). Fourier transform infrared (FT-IR) spectroscopy was collected on an Avatar370 Fourier Infrared Spectrometer (Thermo Nicolet, WA, USA).

### 2.2. Synthesis of MNPs-NH_2_ Nanoparticles

MNPs were prepared by the co-precipitation method reported by Cao et al. [20]. First, 0.02 M of FeCl_3_·6H_2_O and 0.01 M of FeSO_4_·7H_2_O were dissolved in 200 mL of deionized water, and subsequently heated to 85 °C under nitrogen protection with continuous stirring. Then, 10 mL of ammonia hydroxide was rapidly injected into the system, and the mixture was heated for 25 min before cooling to room temperature. The black precipitate was washed repeatedly with ethanol and deionized water, and finally vacuum dried into powder at room temperature.

Then, the Fe_3_O_4_ MNPs were coated with silica by the reverse microemulsion method, as previously described [21], with the following modifications. A reverse microemulsion system was composed of 75 mL of cyclohexane, 18 mL of n-hexanol, 17.7 mL of Triton X100, and 5 mL of water. Approximately 2 mg of dried MNPs was dispersed into the microemulsion and sonicated for about 10 min, and thoroughly stirred for 1 h. Ammonium hydroxide (0.6 mL, 28%) and tetraethoxysilane (TEOS) (1 mL) were then added sequentially to initiate hydrolysis, and the reaction continued for 12 h to produce silica-coated magnetic nanoparticles.

Finally, 1 mL of 3-aminopropyltriethoxysilane (APTES) was added, and the reaction continued for another 12 h at room temperature. The final product (MNPs-NH_2_) was collected magnetically, washed 5 times with ethanol and water, and then vacuum dried to form a powder.

### 2.3. Preparation of the MNPs-NH_2_-C1 Conjugates

In order to prepare MNPs-NH_2_-C1, 1 mL of MNPs-NH_2_ (1 mg/mL) suspension was mixed with 2 mg of EDC and 3 mg of NHS, and stirred for 1 h. Then, the mixture was magnetically separated to remove the excessive EDC and NHS and was dispersed into 50 mM PBS (pH = 7.5) buffer. The addition of 50 μL of C1 (5 μmol/L) to the aforementioned solution was followed by overnight stirring at room temperature. After magnetic separation, the precipitate (MNPs-NH_2_-C1) was dispersed in 1 mL of PBS (pH = 7.5, 50 mmol/L) and stored at 4 °C for later use.

### 2.4. Preparation of AuNPs-GOx-C2 Conjugates

The method in reference [22] was used to synthesize AuNPs. First, 1 mL of 1% (*w*/*w*) HAuCl_4_·4H_2_O was placed in a round-bottom flask, followed by the addition of 100 mL of deionized water. The mixture was refluxed to boiling under vigorous stirring, and then 0.75 mL of 1% (*w*/*w*) sodium citrate was quickly added, changing the color from light yellow to wine red. After cooling to room temperature, it was stored at 4 °C. A 1 mL solution of AuNPs was taken and adjusted to pH 8.0–9.0 with Na_2_CO_3_ (0.01 mol/L), followed by the addition of 400 μL of GOx (1 mg/mL) and 100 μL of C2 (5 μmol/L). The mixture was reacted overnight at 4 °C, centrifuged, and the precipitate (AuNPs-GOx-C2) dispersed in 1 mL of 50 mmol/L PBS (pH = 7.5) at 4 °C for storage and subsequent use [23].

### 2.5. PGM Measurement Toward Target Cu^2+^

First, 100 μL of AuNPs-GOx-C2 was placed into a centrifuge tube, followed by the addition of 100 μL of MNPs-NH_2_-C1 and 100 μL of Cu^2+^ at varying concentrations. Subsequently, 2 μL of 1 mM THPTA and 2 μL of 600 μM sodium ascorbate were introduced. The mixture was incubated at room temperature for 45 min. After incubation, magnetic enrichment was performed, and the precipitate was washed once with deionized water. Next, 50 μL of a glucose solution (20 mmol/L) was added and the sample was incubated at 37 °C for 50 min. Following a second round of magnetic enrichment, 5 μL of the supernatant was placed on a PGM test strip, and the PGM signal value was recorded.

## 3. Results and Discussion

### 3.1. The Principle of of PGM-Based Cu^2+^ Sensing Platform

**Figure 1:**
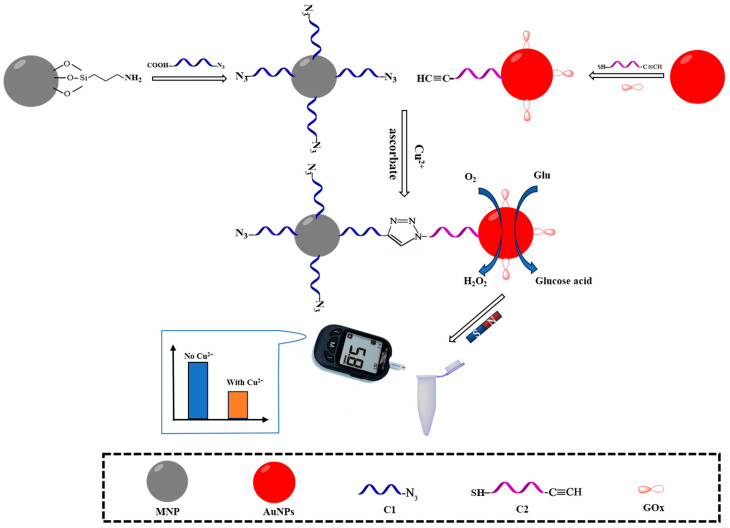
Schematic diagram of Cu^2+^ detection based on portable glucose meter. MNP: Fe_3_O_4_ nano-magnetic beads; AuNPs: gold nanoparticle; GOx: glucose oxidase; Glu: glucose.

The principles of the PGM for Cu^2+^ detection are shown in Figure 1. Two oligonucleotides, C1, which contains an azide group at the 3′ end and is carboxy-modified at the 5′ end, and C2, which contains an alkyne group at the 3′ end and is thiol-modified at the 5′ end, were designed. Firstly, C1 is fixed to the amino-functionalized nanomagnetic bead surface via an amide bond, forming the MNPs-NH_2_-C1 complex. Simultaneously, C2 and GOx are, respectively, covalently decorated on the AuNPs’ surface via Au-S and Au-N bonds, forming the AuNPs-GOx-C2 complex. When Cu^2+^ and a reductant (sodium ascorbate) are present, a Cu^+^-catalyzed azide–alkyne cycloaddition would result in the chemical ligation of C1 and C2, allowing MNPs-NH_2_-C1 to capture the AuNPs-GOx-C2 complex. The GOx on the surface of the AuNPs-GOx-C2 complex catalyzes the oxidation of Glu to gluconic acid and H_2_O_2_, leading to a decrease in Glu concentration in the reaction solution, which is quantitatively monitored using PGM. With the increase in Cu^2+^ concentration, the amount of the AuNPs-GOx-C2 complex captured by MNPs-NH_2_-C1 also increases, resulting in a decrease in Glu concentration in the reaction solution (PGM reading), thus establishing an inverse relationship between the Cu^2+^ concentration and PGM reading.

### 3.2. Characterization of MNPs-NH2-C1 Conjugate

**Figure 2:**
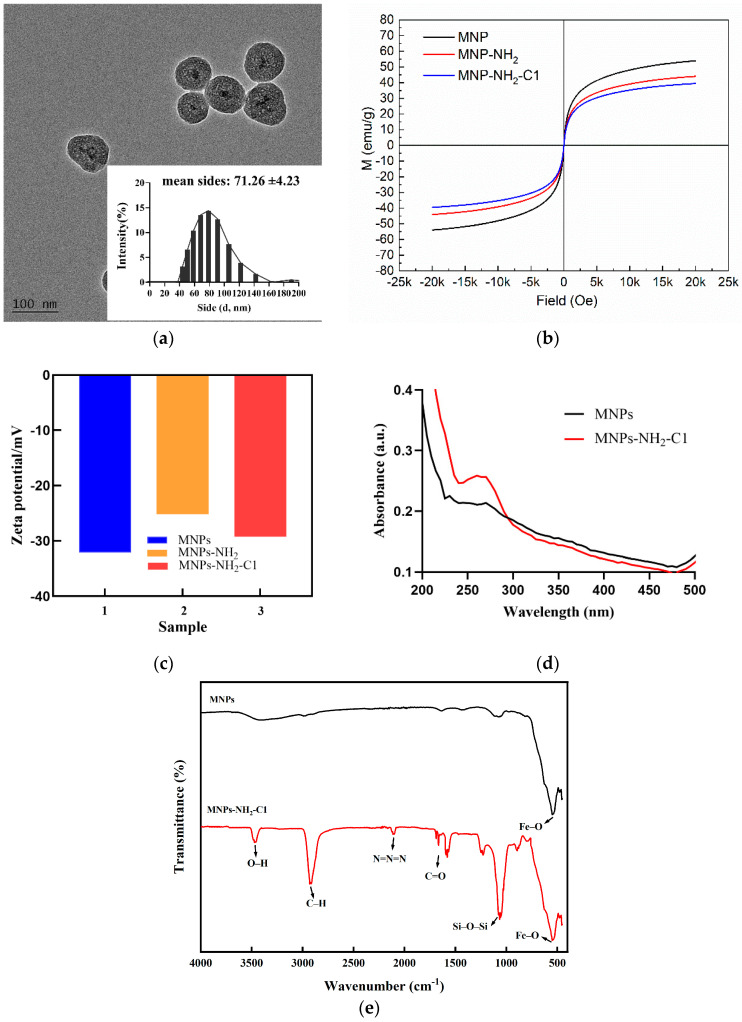
(**a**) TEM image of MNPs-NH_2_-C1, (**b**) VSM magnetization curve of MNPs, MNPs-CH_2_, and MNPs-NH_2_-C1, (**c**) Zeta potentials of MNPs, MNPs-CH_2_, and MNPs-NH_2_-C1, (**d**) UV−Vis absorbance spectroscopy of MNPs and MNPs-NH_2_-C1, and (**e**) FTIR of MNPs and MNPs-NH_2_-C1.

To realize our design, the most fundamental prerequisite for the successful construction of MNPs-NH_2_-C1 is essential. TEM (transmission electron microscopy), Zeta potential, UV−VIS (ultraviolet−visible) absorbance, VSM (vibrating sample magnetometer), and FTIR (Fourier transform infrared spectroscopy) were carried out to characterize the morphology of MNPs-NH_2_-C1 nanoparticles. Figure 2a shows that most of the particles are core-shell and spherical, and the average size of the nanoparticles are 71.26 ± 4.23 nm after modification.

The magnetic property of the MNPs-NH_2_-C1 nanocomposite is an important characteristic for Cu^2+^ detection. The magnetic hysteresis curve of MNPs shown in Figure 2b displays a typical curve for superparamagnetic nanomaterials without any hysteresis. Although silanization, followed by further modification with oligonucleotide C1, could weaken the magnetic saturation value of the sample, the magnetic property of the MNPs-NH_2_-C1 nanocomposite is still strong enough to achieve rapid separation within 3 min using a permanent magnet.

The Zeta potential measurements are shown in Figure 2c, and upon SiO_2_ modification, the magnetic beads exhibited a Zeta potential of −32.1 mV, attributed to the introduction of negatively charged silanol groups (SiO^−^). Following the subsequent modification with amino groups (-NH_2_), the Zeta potential shifted positively to −25.2 mV. The further conjugation of the probe C1 to the MNPs resulted in the Zeta potential returning to approximately −30.3 mV, due to the negatively charged nucleic acid backbone [24].

UV−VIS absorbance spectroscopy was conducted to certify the successful preparation of MNPs-NH_2_-C1. As shown in Figure 2d, MNPs exhibit no significant absorption peaks, and the background signal is weak (curve a). After C1 is modified on the MNPs’ surface through an amide bond, a characteristic absorption peak of DNA appears at 260 nm, which is consistent with the results reported previously [25].

Moreover, the synthesis and modification of MNPs were confirmed by FTIR spectroscopy. As shown in the Figure 2e, the strong absorption band around 580 cm^−1^ arises from Fe–O stretching. The asymmetric stretching vibration of the Si–O–Si bond at 1066 cm^−1^ and the symmetric stretching vibration at 850 cm^−1^ indicate that silica has successfully coated the surface of MNPs through the hydrolysis of TEOS. The absorption band at 2930 cm^−1^ is attributed to CH_3_ in APTES, while the two bands at 3460 cm^−1^ and 1578 cm^−1^ are attributed to the N-H stretching and bending vibrations of free -NH_2_, respectively. These findings suggest that -NH_2_ groups have been bonded onto the surface of silica-coated MNPs through a reaction between –OH and APTES [21]. Furthermore, the characteristic peaks observed between 1600 and 1700 cm^−1^ corresponding to the stretching vibrations of the C=O and C=N bonds in the bases [25], as well as the characteristic absorption peak at 2100 cm^−1^ from the stretching vibration of the azide compound N=N=N group [26], indicate that the azide-modified single-stranded nucleic acids have been successfully modified onto the MNPs’ surface.

### 3.3. Characterization of AuNPs-GOx-C2 Conjugate

**Figure 3:**
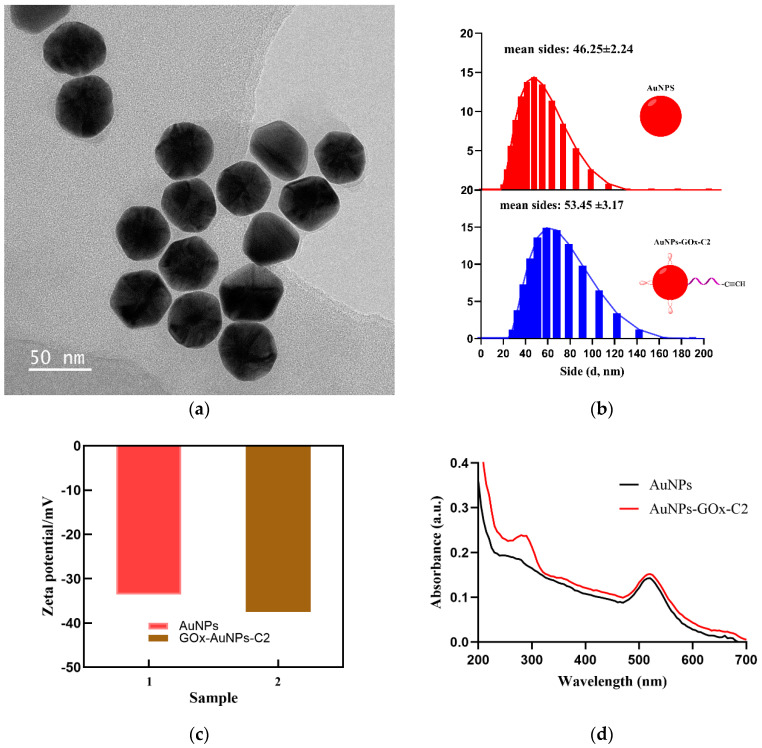
(**a**) TEM image of AuNPs-Gox-C2, (**b**) size distribution of AuNPs and AuNPs-Gox-C2, (**c**) Zeta potentials of AuNPs and AuNPs-Gox-C2, and (**d**) UV−Vis absorbance spectroscopy of AuNPs and AuNPs-Gox-C2.

As described above, the hydrolysis of glucose is mediated by the glucose oxidase immobilized on the AuNPs-GOx-C2 nanocomplex. Therefore, the successful preparation of AuNPs-GOx-C2 is crucial. The transmission electron microscopy image of the AuNPs-GOx-C2 complex, shown in Figure 3a, indicates that the nanostructures of the complex were core-shell and spherical, which provides a precondition for the development of the assay method. Additionally, we used dynamic light scattering to study the size of the gold nanoparticles before and after the reaction with glucose oxidase and C2. As seen in Figure 3b, the average size of the nanoparticles increased from 46.25 ± 2.24 to 53.45 ± 3.17 nm, and the increase in size was due to the labeled proteins, consistent with the results reported in the literature [27]. The Zeta potential of AuNPs-GOx-C2 (−37.5 mV) altered obviously compared to AuNPs (−33.6 mV), demonstrating the successful modification of C2 and GOx with AuNPs (Figure 3c) [28]. To further verify the presence of the proteins on the nanocomplex, we used UV–vis absorption spectroscopy to investigate the AuNPs-GOx-C2. As is well known, the characteristic absorption peaks of gold colloids, proteins, and oligonucleotides are 520 nm, 280 nm, and 260 nm, respectively [29]. As seen from Figure 3d, the synthesized nanocomposite had two distinct absorption peaks at 520 nm and 280 nm. However, possibly due to the relatively less amount of labeled oligonucleotide, we did not observe the characteristic peak of the oligonucleotide [30].

### 3.4. Feasibility Analysis

**Figure 4:**
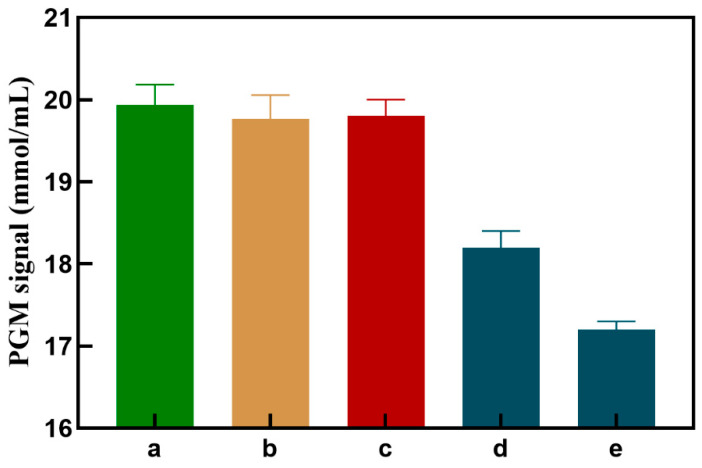
PGM readout signals toward different components: (**a**) Glu, (**b**) MNPs-NH_2_-C1 + Glu, (**c**) MNPs-NH_2_-C1 + AuNPs-GOx-C2 + Glu, (**d**) MNPs-NH_2_-C1 + AuNPs-GOx-C2 + Cu^2+^ (0.1 μmol/L) + Glu, (**e**) MNPs-NH_2_-C1 + AuNPs-GOx-C2 + Cu^2+^ (1 μmol/L) +Glu.

As shown in Figure 4, when MNPs-NH_2_-C1 is present alone, the PGM value (column b) is not significantly different from column a, indicating that MNPs-NH_2_-C1 has no oxidation effect on Glu. When AuNPs-GOx-C2 is added, MNPs-NH_2_-C1 does not undergo a cycloaddition reaction with AuNPs-GOx-C2, and the PGM value (column c) is not significantly changed compared to column a, indicating that GOx-AuNPs-S2 has no significant specific adsorption to MNPs-NH_2_-C1. When different concentrations of Cu^2+^ are present (taking 0.1 and 1 μmol/L as examples), MNPs-NH_2_-C1 and GOx-AuNPs-S2 connect through a cycloaddition reaction, resulting in a significant decrease in the PGM signal, which decreases as the target concentration increases (column d, column e), indicating the feasibility of this experimental approach.

### 3.5. Optimization of the Experimental Conditions

**Figure 5:**
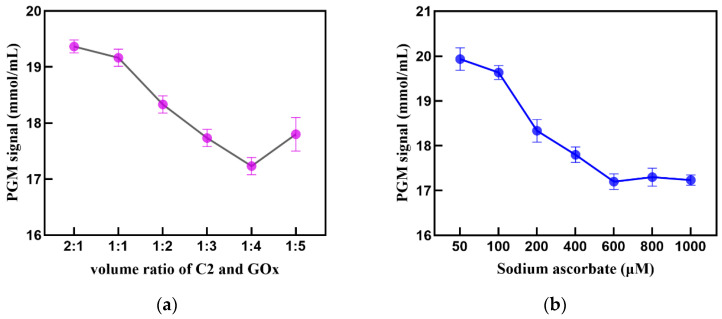

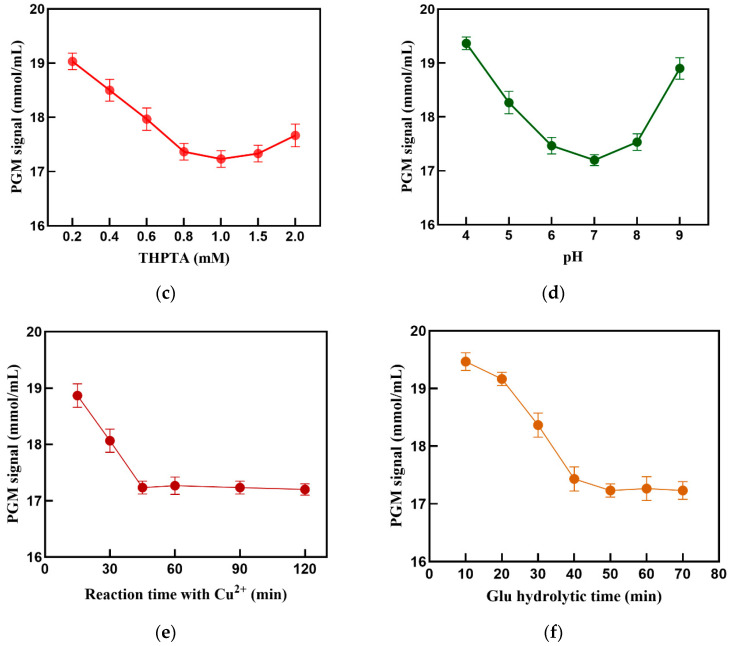
Effect of (**a**) volume ratio of C2 and GOx, (**b**) concentration of sodium ascorbate, (**c**) concentration of THPTA, (**d**) pH value, (**e**) click reaction time, and (**f**) Glu hydrolytic time. The error bar shows the standard deviation (*n* = 3).

Since the PGM response comes from the signal value of Glu oxidation by GOx, it is necessary to optimize the ratio of C2 to GOx labeled on the surface of AuNPs. In theory, a high loading of GOx can enhance the sensitivity of the Cu^2+^ sensor. However, an excess of GOx molecules can affect the cycloaddition reaction between C1 and C2, which is unfavorable for a signal response. As shown in Figure 5a, the optimal volume ratio of C2 to GOx is 1:4 for the PGM response signal. Therefore, 400 μL of GOx (1 mg/mL) and 100 μL of C2 (5 μmol/L) were used to prepare AuNPs-GOx-C2.

The amount of sodium ascorbate, which was used as a reducing agent, and THPTA, which was used to increase Cu^+^ binding, can affect the efficiency of the cycloaddition reaction [31,32]. The relationship between the PGM signal and different concentrations of sodium ascorbate and THPTA were investigated. As the concentration of sodium ascorbate increased, the PGM signal gradually decreased until it plateaued at 600 μM (Figure 5b). Therefore, the optimal concentration of sodium ascorbate was determined to be 600 μM. Meanwhile, a THPTA concentration of 1 mM gave the best sensing performance (Figure 5c).

pH also has a significant impact on cycloaddition reactions [33]. Similar to previous findings [31], the PGM signal remains relatively constant within the pH range of 6.0 to 8.0 and reaches its minimum at pH 7.0 (Figure 5d); hence, pH 7.0 conditions were used in the experiment.

The changing of the PGM signal depended on the yield of the click ligation ssDNA product, which corresponded to the reaction time for the Cu^+^-catalyzed cycloaddition. As shown in Figure 5e, the minimum PGM signal occurs at 45 min, with little change thereafter. Therefore, 45 min was chosen as the optimal reaction time.

Glucose hydrolysis is a key factor affecting the PGM signal in the detection system, and the reaction time directly impacts the detection results. As shown in Figure 5f, when the reaction time is less than 50 min, the PGM signal gradually decreases with the increasing reaction time; after exceeding 50 min, the signal stabilizes. Therefore, the optimal hydrolysis time for glucose is 50 min.

### 3.6. Detection of Cu^2+^ Under the Optimum Experimental Conditions

**Figure 6:**
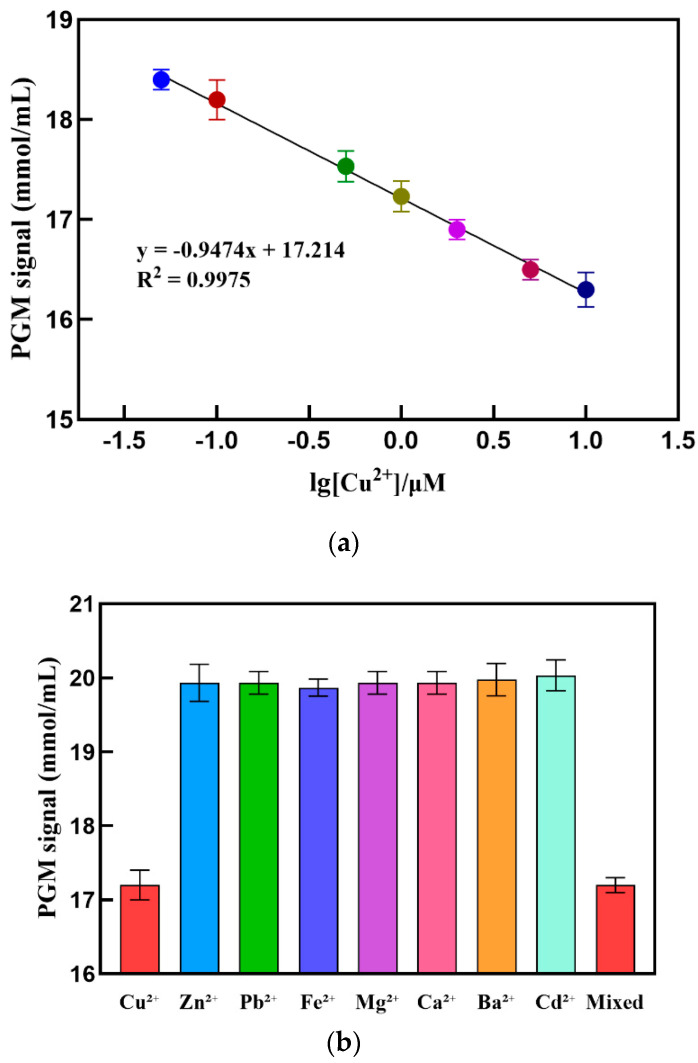
(**a**) Linear calibration curve for detection of Cu^2+^; (**b**) specificity test result, 1 μmol/L of Cu^2+^, 1 μmol/L of Zn^2+^, Pb^2+^, Fe^2+^, Mg^2+^, Ca^2+^, Ba^2+^, and Cd^2+^, respectively, and a mixture of these ions mixed together.

Under the optimal experimental conditions, the response signal of different concentrations of Cu^2+^ was determined using PGM. As shown in Figure 6a, the PGM signal gradually decreases with increasing concentrations of the target substance Cu^2+^. Within the range of Cu^2+^ concentrations from 0.05 to 10.00 μmol/L, there is a good linear relationship between the PGM signal and the logarithm of Cu^2+^ concentration, with a linear equation of y = −0.95x + 17.21 and an R^2^ value of 0.9975. The detection limit is 0.03 μmol/L (3σ). This detection limit is lower than the WHO’s (∼30 μmol/L or 2 ppm) [34] and US EPA’s (∼20 μmol/L or 1.3 ppm) [35] maximum allowable amount of Cu^2+^ in drinking water. In addition, compared to the methods for detecting Cu^2+^ reported in Table 1, this method exhibits significant superiority.

Moreover, as shown in Figure 6b, only Cu^2+^ caused a significant decrease in the PGM signal, while other interfering ions at the same concentration (Zn^2+^, Pb^2+^, Fe^2+^, Mg^2+^, Ca^2+^, Ba^2+^, and Cd^2+^) did not cause any noticeable signal change, indicating that this method has good selectivity for Cu^2+^. Subsequently, the relative standard deviations for detecting 0.1, 0.5, and 5.0 μmol/L of Cu^2+^ were 0.8%, 1.2%, and 0.9%, respectively, indicating the good reproducibility of this method.

### 3.7. Detection of Cu^2+^ in Real Samples

Tap water samples were selected for testing the potential application of our fabricated biosensor. As shown in Table 2, the spiked recovery rates for Cu^2+^ at different added concentrations ranged from 92.30% to 113.33%, with an RSD of 0.14% to 0.34%, indicating excellent practical application ability in real analytical samples.

## 4. Conclusions

In summary, a novel method for the detection of Cu^2+^ was developed based on the PGM platform and Cu^+^-catalyzed click chemistry. This approach offers a portable, sensitive, selective, and quantitative means of Cu^2+^ detection, utilizing the widely accessible PGM. The method relies on the Cu^+^-catalyzed alkyne–azide cycloaddition reaction to convert Cu^2+^ concentration into a detectable PGM signal, achieving a limit of detection (LOD) of 0.03 μmol/L. When applied to tap water samples spiked with Cu^2+^, the method demonstrated high recovery and accuracy. Due to the portability, simplicity, and broad availability of PGM, we expect that the developed method will be a promising new tool for trace Cu^2+^ analyses, and will find wide application in field-based testing and even for families.

## Figures and Tables

**Table 1:** Comparison of different sensing methods for detection of Cu^2+^.

Method	Sensor Content	Linear Range	Detection Limit	Ref.
Colorimetric	AuNPs/DNA probe	0.5–10 μmol/L	0.25 μmol/L	[33]
Colorimetric	P-AuNPs	20–60 μmol/L	0.43 μmol/L	[36]
Fluorescence	DNA/graphene oxide(GO)	0.1–10 μmol/L	0.058 μmol/L	[37]
Fluorescence	carbon dots/o-phenylenediamine	1–100 μmol/L	0.329 μmol/L	[38]
Chemiluminescence	red carbon dots	1–50 μmol/L	0.619 μmol/L	[39]
Chemiluminescence	sulfur and nitrogen co-doped carbon quantum dots	0.15–7.8 μmol/L	31.5 μmol/L	[40]
Chemiluminescence	CdTe quantum dots/K_3_Fe(CN)_6_	0.12–900 μmol/L	100 μmol/L	[41]
Glycemic analysis	MNPs-NH_2_-C1/AuNPs-GOx-C2	0.05–10.00 μmol/L	0.03 μmol/L	This work

**Table 2:** Determination results of Cu^2+^ in tap water samples.

Sample No.	Added/(μmol/L)	Found/(μmol/L)	Recovery (%)	RSD (%)
1	0	0	—	—
2	0.1	0.09	92.30	0.24
3	1	1.06	105.51	0.24
4	5	4.86	97.10	0.14
5	10	11.33	113.33	0.34

## Data Availability

The original contributions presented in the study are included in the article. Further inquiries can be directed to the corresponding author.
